# Geolocated fish spawning habitats

**DOI:** 10.1038/s41597-024-03348-3

**Published:** 2024-05-22

**Authors:** Kimberly L. Oremus, James Rising, Nandini Ramesh, Audrey J. Ostroski

**Affiliations:** 1 University of Delaware, School of Marine Science and Policy, Newark, DE 19716 USA; 2 Commonwealth Scientific and Industrial Research Organisation, Natural Systems Modelling Group, Data61, Eveleigh, NSW 2015 Australia

**Keywords:** Biogeography, Ecology

## Abstract

Fish spawning locations are a crucial input into fisheries management and conservation plans, and many stocks are especially sensitive to the environmental conditions within these localized zones. Globally collated data on spawning locations across many species has been unavailable, hindering global stock assessments and analyses of sustainable development and global environmental change. To address this, we created a geocoded fish spawning dataset using qualitative spawning information from FishBase and Science and Conservation of Fish Aggregations (SCRFA). We cleaned and geocoded the spawning locations of 1,045 marine fish species into 2,931 regions. Each spawning region is defined by one or more polygons, and most spawning regions are associated with spawning months. The resulting dataset covers oceans globally. This dataset will be useful to scientists studying marine fish population dynamics and their interactions with the physical environment on regional to large scales.

## Background & Summary

Marine capture fisheries make up 97 million tonnes of global fish production, provide 8% of global animal protein consumption, and generate USD 151 billion in global revenue annually^1^. However, sustainable management remains a challenge for many species, particularly under the risks of climate change^2–4^.

Geospatial data has emerged as a powerful tool to support research in this area, spurred by new data products. These data products monitor fishing activity using satellites^5^; variations in oceanographic environmental conditions using satellites^6–9^, drifters^10^, and moorings^11–14^; and biological activity using remote operating vehicles^15^, SONAR^16^, and fish tagging^17^. However, not all biological activity can be detected with these monitoring systems, and this is especially the case with reproduction and spawning. These stages of the life cycle are particularly sensitive to environmental stressors, which are projected to increase under climate change^18–20^. Existing literature emphasizes the need to better understand these vulnerable life stages of fish to improve marine capture fisheries management^21–23^.

An important motivation for this dataset’s global scale and spatial resolution is its compatibility with data derived from global climate and Earth system models. These have become an indispensable tool for examining and predicting the impacts of global environmental change. These models have increasingly sought to assimilate and simulate biogeochemical variables and processes^24^, and they now simulate a large number of variables that describe the physico-chemical environment in which marine fish live. More recent iterations include not only physical variables such as temperature, salinity, and current velocities, but also chemical and biological factors like pH, oxygen levels, and chlorophyll concentrations. The ability to pair simulated conditions from these models with data on the timing and locations of spawning^20,21^ offers new avenues for examining the potential impacts of climate change on marine fish species.

This dataset^25^ fills this gap by providing global coverage across all major assessed species. The spatial resolution is approximately 0.5° × 0.5°, higher than that of most Earth system models used in the Coupled Model Intercomparison Project (1.5–2 degrees)^26^ and comparable to that of popular reanalysis datasets^27–30^. It could therefore be used in combination with these to analyze a variety of ecosystem interactions in the context of climate change projections. In combination with other geolocated data, such as data on marine protected areas^31^, fishing activity^5^, and country-level fish catch and stock assessments^1,32–34^, this dataset will help answer key scientific questions regarding the location of critical habitats, the role of transboundary spawn flows, and emerging risks under global environmental change. These insights can aid in the creation of policy solutions.

**Fig. 1:**
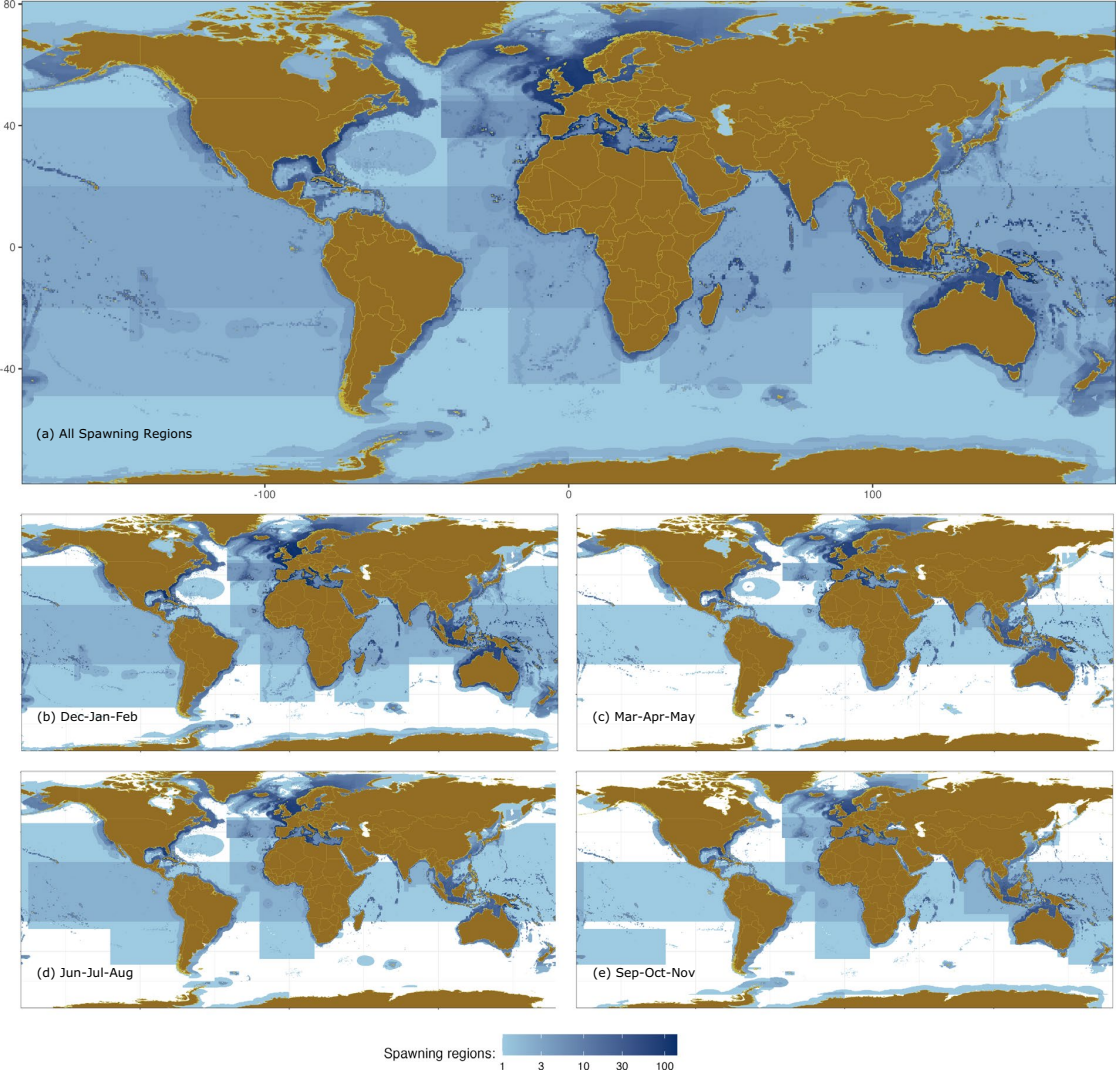
Number of overlapping spawning region records (polygons or groups of polygons). Counts are provided (**a**) in total, and number of spawning region records by season: (**b**) northern winter/southern summer (December-February), (**c**) northern spring/southern autumn (March-May), (**d**) northern summer/southern winter (June-August), and (**e**) northern autumn/southern spring (September-November). Colors follow a log scale. Some spawning regions exhibit long, straight edges, which are variously produced by corresponding edges within the AquaMaps’s native range data^37^, FAO Regions^39^, and manual or geocoded boxes.

We geocoded spawning regions for 1,045 marine fish species described in the FishBase^35^ and Science and Conservation of Fish Aggregations (SCRFA)^36^ public datasets. These global databases have painstakingly aggregated the fieldwork of countless biologists and ecologists to summarize our knowledge of fish species. We further constrained geographic locations using AquaMaps^37^ to produce 2,931 polygons or groups of polygons, which we call “spawning regions” (Fig. 1). Spawning regions range from 1.9 × 10^−3^ km^2^ to 1.3 × 10^8^ km^2^ with a median of 8.5 × 10^4^ km^2^.

## Methods

**Fig. 2:**
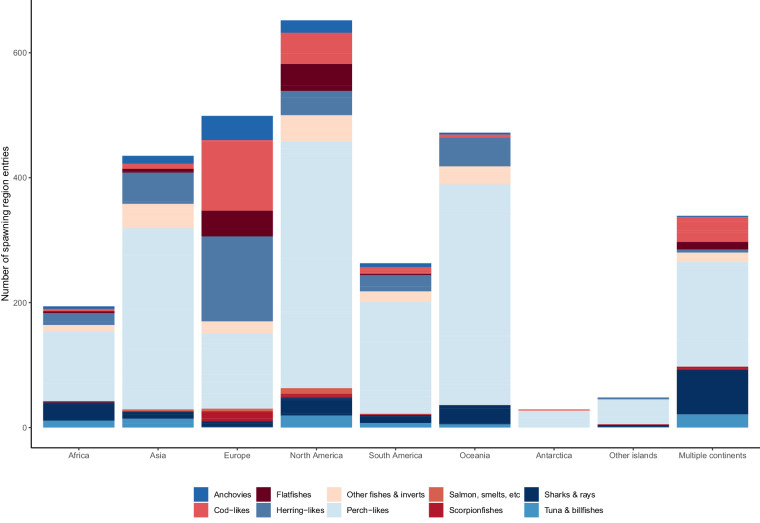
Number of spawning regions by continent and commercial group. Continent definitions are drawn from Natural Earth^45^. “Multiple continents” indicates that the spawning region record is located across two continents or more (such as the Mediterranean Sea) and can include the high seas. Records in the “Other islands” category are located in island groups outside of Oceania and not in close proximity to a continent.

A list of commercially caught fish species from 1950–2014 was collected from Sea Around Us^32^ (Fig. 2). The spawning location records from FishBase^35^ were collected for these 1,045 species groups and combined with the SCRFA^36^ spawning aggregation locations. A single spawning observation in either of these databases was sufficient to include that species. In FishBase, spawning information is provided for many species, with attributes describing the country, locality, and spawning months for each of multiple observed spawning locations. The description of locality varied in specificity from highly descriptive terms such as “Wemindji, eastern James Bay (1987–1988)” to “North Atlantic.”

To construct spawning regions, we first created polygons based on spawning locality and species information. To construct these spawning locality polygons, we used one of six methods (described below): Exclusive Economic Zone (EEZ), FAO Region, Automated Geocoding, Manual Geocoding, Native Range, and Marine-Feature Polygon. We used a spreadsheet to keep track of each spawning location, its associated species groups, and the coding method. This file, Master Spawning ProCreator.csv, can be found in the GitHub repository described under Data Availability. Our process for deciding which method to use for each species is explained below in the descriptions of the coding methods. Second, we intersected the locality polygons with the species’ native range and occurrence records (point map) from AquaMaps^37^, available at a 0.5° resolution. AquaMaps defines the native range as “all areas with suitable environmental conditions that fall within a species’ natural distributional range, as known from the literature.” The point map is a gridded, georeferenced map of occurrence records by species, aggregated from several databases including FishBase.

Overlapping areas between our locality polygons and AquaMaps’ native range and point maps were treated as spawning regions. Though AquaMaps produces a suitability metric for the native range, we assumed all areas with non-zero suitability were equally suitable for spawning. We also treated a locality polygon as a spawning region if it was smaller than three half-degree grid cells, even if it was not part of its native range or point map. However, larger locality polygons that did not overlap with any part of the native range or point map were removed out of caution to avoid potentially misattributing a large spawning region. If there was no native range available in AquaMaps, the record was also removed from the dataset. Finally, if the locality polygon was located over an area that was largely covered by land, as in the case of a river, fjord, or estuary, we set the spawning region to the closest marine grid point within 100 km that was classified as suitable for that species.

All spawning regions are shown in Fig. 1a, and spawning regions with paired monthly spawning data are shown in seasonal Fig. 1b–e. If the record had no associated spawning months with the locality or country, the spawning region is only included in Fig. 1a. Details of the method used for each geocoding category are described below. Only eight spawning regions had to be geocoded using more than one method.

### EEZ

If the “Country” field is given and the “Locality” was missing or does not describe a sub-region, then we used the EEZ polygon(s)^38^. This includes localities that are blank, NA, “along the entire coast,” “off [country name],” the name of the country, or “to be filled.” We used the EEZ polygon(s) for 909 spawning regions.

### FAO region

Of the spawning region records, 373 were larger than the EEZ of one country and were coded as “FAO Region.” This refers to a cross-reference table that matched the locality to Food and Agriculture Organization (FAO) fishing areas and subareas. An example is the Baltic Sea. We matched the locality to the FAO fishing area or subarea polygons^39^. For 10 spawning regions, some of the descriptions (such as “areas poleward”) required us to limit the FAO-defined spawning region by latitude. We used 35° S - 35° N for the subtropics and tropics, 23.5° S - 23.5° N for the tropics, 20° S - 20° N for the equatorial region, and 30° S - 30° N for lower latitudes. If a locality could not be matched to an FAO fishing area(s) or subarea(s), we searched for an existing shapefile that matched the description (see Marine-Feature Polygon) or manually geocoded the area (see Manual Geocoding).

### Automated geocoding

Most of the spawning localities were more descriptive than the EEZ or FAO Region, such as “Gulf of Maine, USA.” We first used two automated geocoding services that provide information on the bounding box of a location, provided by name: ArcGIS World Geocoding Service (https://geocode.arcgis.com/) and GeoNames Search Webservice (https://www.geonames.org/). For each region, we queried each service for “[Country], [Localities],” for the country and localities referenced in the spawning data. The results of these searches were reported by the services as either geolocated points or bounding boxes (rectangles). We selected the polygon that best matched the record’s locality description. Additional details for the geocoding searches are provided below.

#### GeoNames search webservice

Permitted search results were limited to the following feature classes (class code in parentheses): administrative regions (A), water bodies (H), parks and reserves (L), named spots (S), geographic features (T), and undersea features (U). If a result was found without a bounding box, a square 0.5° in size centered on the location was used. A total of 132 spawning region records were coded this way. (Documentation for this service can be found at https://www.geonames.org/export/web-services.html).

#### ArcGIS world geocoding service

Confidence and quality measures are reported for the service, but not used in the manual geocoding process. Forty-nine spawning region records were coded this way. (Documentation for this service can be found at https://developers.arcgis.com/documentation/mapping-apis-and-services/geocoding/).

### Manual geocoding

Only 181 spawning regions could be geocoded using the automated method above. For the remaining localities that were either smaller than the EEZ, such as “Eastern Cape coast, South Africa,” or spanned multiple EEZs, such as “Northeastern Caribbean,” a box was manually described. In some cases, multiple boxes had to be drawn to ensure coverage of the described area. A total of 1,236 spawning region records were manually geocoded.

### Native range

In rare circumstances (only four in the entire dataset), the species was said to spawn wherever it is found. We recorded these as having a spawning range that covered its entire native range. We extracted the present day “Native Range” grid cells from AquaMaps^37^. For 19 spawning regions, more detail was given such as “In equatorial waters” or “Northern hemisphere.” In these instances we clipped the native range to bands of latitude. We used 23.5° S - 23.5° N for the tropics, 20° S - 20° N for the equatorial region, and 30° S - 30° N for lower latitudes.

### Marine-feature polygon

When available, we used shapefiles of seamounts^40,41^ and well-known marine features^38,42^. In the situations where the locality was described as nearshore, such as “shores of Senegal,” estuaries, or ports, we used a polygon 25 km from the coastline as the spawning location. If some indication of the continental shelf or insular slopes was mentioned, we visually checked the native range to see if it included the continental shelf or insular slopes. If the locality referred to depth, such as shelf, upper insular slopes, continental slope, or deep waters, we used bathymetry data^43,44^ to clip the EEZ polygon to that depth. We defined the continental shelf as having a depth of 200 meters (m) or less. Slopes were defined as having a depth between 200 and 3,000 m. We coded 201 spawning regions using at least one of these marine features.

### Dropped records

If the species could not be found in FishBase^35^ or SCRFA^36^, it was removed. If the record did not have a “Locality” and “Country,” it was removed from the dataset. This included records that were blank, “NA,” or “Not specified.” Records were retained if they had either a “Locality” or “Country.” If the species was a fresh water or diadromous fish or the locality was a river, the record was removed. If the record had locality listed as an aquarium, aquaculture site, or lab, it was removed.

## Data Records

The dataset, Geolocated Ocean-Fishery Identified Spawning Habitats (GO-FISH), is available at Zenodo^25^ and consist of features stored in an ESRI Shapefile, along with associated spawning information. Each feature, described by a polygon (or group of polygons), corresponds to a unique species-region combination. Some regions are associated with multiple species, and these regions are included multiple times (often with different spawning month information). Most species have multiple spawning regions, and these are included as separate polygons (again, often with different spawning months). Table 1 describes the information attributes associated with each polygon.

**Table 1:** Variable overview of GO-FISH shapefiles.

Variable Name	Description
PID	The spawning region ID.
X1-X12	Spawning month variables, where X1 is January and X12 is December. Values between 0 (no spawning reported) and 100 (spawning reported) are reported for each month, with intermediate values representing the monthly percentage of mature females. This data is provided by FishBase^35^.
species	The species name.
country	The country (if the spawning region is a national or subnational unit).
localities	The locality, where available. If the country is missing, the locality refers to a supranational region.
source	Source of the qualitative spawning region description, which is either FishBase^35^ or SCRFA^36^.
method	The geocoding method employed, described above.

## Technical Validation

While it is not possible to re-collect field observations on spawning locations or reread all the primary literature from FishBase^35^ and SCRFA^36^ to validate their spawning “Country” and “Locality,” we performed the following data checks.

If the locality description was ambiguous (e.g., “North Atlantic”), we considered the species range and FishBase or SCRFA description to provide a clearer regional definition. In 38 cases, the same region description is interpreted differently based on the species.

If the country and locality description did not appear to be consistent, we considered the species range and FishBase or SCRFA description to update the information. This resulted in 56 changes, of which in 19 a country was added, in 14 a country was removed, and in 23 the country was changed.

If the spawning region crossed the international dateline, we created two separate polygons to ensure the resulting spawning region was unambiguous.

If the locality polygon did not overlap with the AquaMaps-defined native range of the species^37^, we hand-checked those records. In some cases, we had to go back to the primary source that FishBase listed. In two cases, the record was dropped because we uncovered human error between FishBase and the primary resource that FishBase cited.

Finally, all records without a country listed were checked by two individuals, as these records were not necessarily bounded by a country’s EEZ. The remaining records were spot checked by a second individual.

## Usage Notes

The GO-FISH dataset^25^ contains geospatial spawning locations by species from FishBase^35^ and SCRFA^36^.

- This dataset is not a comprehensive dataset of spawning locations and should not be used to represent all global spawning. The records are limited to commercial fish species. The data is more abundant in locations that have a long history of biological research activities. There is less data in the high seas and in less-studied locations (Fig. 1).
- GO-FISH is derived from qualitative descriptions from a variety of field research, not standardized observations with recorded latitude and longitude. The descriptions range from ambiguous (e.g., “North Atlantic”) to specific (e.g. “Wemindji, eastern James Bay (1987-1988)”). To avoid misattributing large spawning locations, we limit these spawning regions to those that intersect with a species’ native range or occurrence data in AquaMaps^37^. However, not all of a species’ native range may be suitable for spawning, which means the dataset may still misidentify some non-spawning locations as spawning locations (false positives). At the same time, species with specific and small spawning location descriptions may spawn in places not represented by this dataset (false negatives). A careful read through the methods section for all the assumptions is warranted. We recommend this data be used for analyses that have a 0.5° × 0.5° resolution or lower.
- Some polygons will have long, straight edges due to the geocoding method. For example a record coded as “Native Range” with a locality of northern hemisphere will have a straight edge along the 0° latitude. Other geocoding methods that produce straight edges include some FAO Regions, manual or geocoded boxes, and constraining the spawning location with AquaMaps’s suitability data^37^.
- The dataset is not attached to a year and therefore cannot reflect changes to spawning locations over time. However, the data is paired with the monthly spawning periods found in the “Spawning” data category in FishBase. We recommend using mean seasonal cycles (climatologies) over a span of 10 or more years when pairing this data with physical environmental datasets, such as sea surface temperature.

## Data Availability

The scripts for constructing the inputs to the dataset and generating the dataset shapefile, data outputs, and figures are in https://github.com/openmodels/spawning-dataset. This repository also contains the necessary input files, intermediate outputs, and final outputs. Instructions for reproducing the dataset are provided in the README.md file.
